# HIV risk behaviours among women who inject drugs in coastal Kenya: findings from secondary analysis of qualitative data

**DOI:** 10.1186/s12954-019-0281-y

**Published:** 2019-02-06

**Authors:** Gitau Mburu, Mark Limmer, Paula Holland

**Affiliations:** Division of Health Research, University of Lancaster, Lancaster, LA1 4YW UK

**Keywords:** HIV, Injecting drug use, Female, Heroin, Qualitative, Kenya

## Abstract

**Background:**

Injecting drug users are at high risk of HIV infection globally. Research related to female drug users is rare in Kenya, yet it is required to inform the development of gender-sensitive HIV prevention and harm reduction services in East Africa, where injecting drug use is on the rise.

**Methods:**

This study aimed to document the nature of HIV risks encountered by women who inject drugs in the Mombasa and Kilifi, Kenya. Secondary data analysis was conducted on an existing dataset from a 2015 primary qualitative study involving 24 interviews and 3 focus group discussions with 45 women who inject drugs. These were complemented with five interviews with key stakeholders involved in the provision of services to women who inject drugs. Guided by the social ecology theory, a thematic analysis was conducted to identify the nature of HIV risks and their underlying determinants.

**Results:**

HIV risk behaviours fell into two broad categories: unsafe injecting and unprotected sex. These risks occurred in the form of sharing of needles, unprotected oral, anal, and vaginal sex, sexual assaults, injecting drug use during sex, sex work, and other types of transactional sex. The primary determinants underlying these risks were a low-risk perception, inequitable gender power, economic pressures, and poor availability of needles and condoms. These social-ecological determinants did not exist in isolation, but intersected with each other to create powerful influences which exposed women to HIV. Social-ecological determinants exerted constant influence and created a persistent ‘HIV risk environment’ that was involuntarily experienced by women.

**Conclusion:**

Individual, interpersonal, and societal-structural factors intersect to produce HIV risk behaviours. As a minimum, these risks will require a combination of multifaceted micro-level interventions including self-efficacy training, risk assessment skills, couple counselling, and universal access to the recommended harm reduction package. In addition, the current focus on micro-level interventions in Kenya needs to shift to incorporate macro-level interventions, including livelihood, employability, and gender norms-transforming interventions, to mitigate economic and gender-related drivers of HIV risks. In the Kenyan context, injecting drug use during sex work is emerging as an increasingly important HIV risk behaviour needing to be addressed.

## Background

Kenya is one of the countries most affected by HIV globally [1]. It has a generalised HIV epidemic and a national prevalence of 5.6% [2]. There are 1.6 million people living with HIV nationally, majority of whom acquired it via unprotected heterosexual sex [3, 4]. An additional 100,000 new HIV infections occur annually, 80% of these being among adults [4]. Despite these stark statistics, epidemiological data shows that HIV prevalence is on a decline in Kenya, having peaked at a prevalence of 10.5% in the late 1990s [4]. Similarly, HIV incidence has been on a decline from an annual peak of 116,000 infections in 2009 [4].

In order to sustain these gains in HIV epidemic, emerging epicentres of high incidence will need to be continually addressed. For instance, although heterosexual sex has historically been the main driver of HIV epidemic in Kenya, injecting drug use is emerging as an important source of new HIV infections. The first modes of HIV transmission study in Kenya to include injecting drug users was conducted in 2008. It found that injecting drug use contributed 3.8% of HIV cases nationally [3]. This relatively low contribution of injecting drug use to the national HIV prevalence has contributed to an inadvertent neglect of people who inject drugs (PWID) in Kenya [5]. However, the importance of injecting drug use comes to the fore when HIV infection within PWID themselves is considered. For instance, recent modelling studies estimated that 18.3% of the 18,327 injecting drug users nationally were infected with HIV [4, 6]. This prevalence is more than three times the national rate [2].

A number of other recent studies have supported the view that injecting drug use is rapidly rising in Kenya and other parts of East Africa [7–9]. In their review of 21 high HIV epidemic countries globally, Petersen et al. [10] concluded that ‘while injecting drug use is relatively rare in sub-Saharan Africa, it is the main driver of HIV in Mauritius and Kenya’. Indeed, the high prevalence of HIV among Kenyan PWID is similar to that reported among other PWID globally [11], suggesting that Kenya is indeed becoming an epicentre of drug use-related HIV epidemic. The significance of injecting drug use in driving the Kenyan HIV epidemic particularly manifest at the coast where nearly half of all PWID live, and 20.5% of these are infected with the virus [12].

The rise in injecting drug use could further accelerate HIV transmission in Kenya, where HIV is already widespread among heterosexual adults, particularly women. In response to the phenomenon of injecting drug use, the Ministry of Health introduced a harm reduction approach within the National HIV/AIDS Strategic Plan in 2013 [13], largely comprising the provision of needle and syringe exchange program (NSP), HIV testing, and opioid substitution therapy (OST) [4]. These interventions are based on the World Health Organization’s recommendations for a package of services for all PWID globally [13]. This package includes needles and syringes, opioid substitution therapy (OST; also referred to medically assisted therapy), drug overdose treatment, HIV testing, antiretroviral therapy (ART), condoms, health education, and treatment of sexually transmitted infections (STIs), tuberculosis (TB), and viral hepatitis [14].

Despite the importance of gender in determining harms of drug use [15], and the increasing emphasis on injecting drug use within the national HIV program, relatively little data exists related to social contexts of injecting drug use among women [16]. In Kenya, elaborating the vulnerabilities to HIV among female drug users is particularly pertinent as women are generally more affected by HIV [4]. Overall, 6.9% of Kenyan women are infected with the virus compared to 4.4% of men [17]. In this context, women are inequitably affected by HIV partly due to gender inequalities and other socio-cultural determinants such as early sex debut [18], gender-based sexual violence [19], and economic inequality in a patriarchal society [20].

Despite women being inequitably affected by HIV nationally, females that injecting drugs—a practice that is a known risk of HIV [11]—have been relatively understudied. Although a number of investigators such as Guise et al. [21], Rhodes et al. [22], and Kurth et al. [12] have explored injecting drug use in Kenya, their study samples were predominantly male (74%, 70%, and 85% respectively). Similar to the global situation [23], much of what is known about injecting drug use locally has been generated from male drug users. Cognisant of this gap, Guise et al. [21] has called for studies to be conducted, exploring the ‘vulnerability of women, and how drug-related harms are gendered or structured by gender relationships in this context’. This kind of data is needed to inform gender sensitive interventions for preventing HIV and other harms of injecting drug use among women. This is a current need, given that harm reduction programs are currently in development in much of East Africa [24]. Given the foregoing, the aim of this paper is to (1) document HIV risks among women who inject drugs in coastal Kenya and (2) discuss potential implications for services, policy, and future research.

## Methods

### Primary study aims and design

This paper reports information generated through a secondary analysis of pre-existing qualitative data. The primary study [25, 26] was a qualitative study conducted in 2015 with aim of exploring the needs, barrier, and determinants of sexual and reproductive health (SRH) of women who inject drugs in the Kenyan coastal towns of Mombasa and Kilifi.

### Aims, setting, and methodology of primary data collection

Data were collected through a combination of focus groups and interviews. Participants comprised of 45 women who inject drugs and 5 key stakeholders involved in the provision of services to people who inject drugs. Of the 45 women, 24 participated in interviews (12 in each site) and 21 participated in three focus groups sessions (2 sessions in Mombasa and 1 session in Kilifi). To be eligible, women had to be adults aged at least 18 years in order to provide independent consent; be within the reproductive age bracket of 18–49 years; and have injected drugs within the past 90 days, which was the definition of current injecting drug use in this study.

The above participants were recruited through convenient sampling via two community-based organisations (CBO). These CBOs were providing harm reduction services through a community-based outreach model whereby injecting drug users were provided with interventions in their own localities and neighbourhoods, or through walk-in services at drop-in centres. Through this model of service provision, outreach workers and drop-in-centre staff provided injecting drug users with clean needles, syringes, drug addiction counselling, HIV counselling and testing, condoms, and a range of essential reproductive services, such as pregnancy tests and contraceptive pills. Injecting drug users that required additional services were referred to nearby government services.

Women were approached and invited to participate by outreach workers during outreach, or by staff at drop-in centres. Recruitment of participants was informed by saturation of data as specified in the primary study protocol. Prior to data collection, women were informed of the aim of the study and required to provide written consent. In the IDI and FGDs, women were asked to describe their needs related to their sexual and reproductive health, their drug use, HIV testing, and access to community and facility-based health services. At the end of the IDIs and FGDs, women were asked to fill a short questionnaire regarding their age, main source of income, marital/relationships status, drug use by their regular partner, condom use, HIV testing, contraception, prior experience of physical or sexual abuse, incarceration, and other social-economic characteristics. To complement perspectives from women who inject drugs five stakeholders who were experienced in providing services to people who inject drugs were selected to participate in interviews related to the above topics. The five stakeholders, three of whom were women, included a community health worker (*n* = 1), outreach workers (*n* = 2), a ministry of health official (*n* = 1), and an outreach manager (*n* = 1). These stakeholders were recruited purposely in collaboration with the two community-based organisations based on their expertise in community outreach, government policy, and harm reduction service resourcing and management. All IDIs and FGDs were conducted face-to-face at the CBOs or stakeholders’ offices either in Swahili or English, based on participants’ preference. In addition, IDIs and FGDs were audio-recorded and lasted between 45 and 60 min. Additional details regarding the primary data collection and analysis have been reported in a series of several primary manuscripts [16, 25–27].

### Social demographic profile of primary participants

The social demographic profile of the sample has been published the above primary papers [16, 25–27]. In brief, the sample was relatively young (mean age 28.5 years), and poorly educated, with a fifth (18%) not having had formal education. Over half (53%) were single, while the rest either had a live-in partner (27%) or were married (18%). Most relied on sex work (29%), ‘hustling’, or casual labour for income. Over a quarter (27%) were homeless and over half (53%) had a history of being imprisoned. Most had at least one child, and did not use contraception, nor attend antenatal care when they got pregnant [25, 26]. Their access to sexual and reproductive health services was prevented by user fees, transport costs, and stigma [16, 25].

### Secondary data analysis

The entire dataset from the primary sample was subjected to secondary analysis, based on a set of new secondary research questions, related to drug use and associated HIV risks. Using the primary dataset, drug use, injecting, and sexual behaviours were summarised. Transcripts were imported into Nvivo® (QSR International) [28], and emerging codes related to injecting or sexual risk behaviours identified guided by a theoretical framework, namely the social ecological model [29]. Codes were refined by continuously examining text segments for similarities and differences [30] and refined codes then organised to identify overarching descriptive and analytical themes [30]. Coding was conducted by one author (GM).

### Theoretical approach: the social ecology theory

Given the observation that ‘interventions are more likely to be effective if they are theory based—namely, if they draw upon a theoretical underpinning of the established determinants of behaviour’ [31]—this study gave emphasis on theoretical elaboration of the nature of HIV risks to increase the likelihood that potential interventions would be effective. This is particularly relevant given that harm reduction programs are currently in development in Kenya, and indeed, much of East Africa [24]. Specifically, this study uses the social ecology theory, which hypothesises that health behaviours are determined by people’s interaction with their physical, social, and structural environments [32]. In his seminal work, Bronfenbrenner [33] suggested that individual health and development was influenced by what he labelled micro, meso, and exo environments. The social ecological model is illustrated as concentric circles each inside the next, representing successive domains of the social ecology [34]. Historically, the social ecology theory emerged in response to a critique of health promotion services that over-relied on individual-level interventions while failing to account for wider social determinants of health [32].

Recently, similar critique has also been levelled against HIV prevention programmes. According to Baral et al. [35], theory-based elaboration of HIV risks tends to focus on personal and micro-contexts, ignoring macro-determinants of HIV-related risky behaviours. Consequently, Baral et al. [35] argue that social-ecological approach is required, especially for key populations, whose HIV risks are affected by structural forces. While useful, popular health promotion theories such as social cognitive theory, health belief model, and theory of reasoned action do not necessarily take into account the availability of social resources, and other protective factors situated within communities and how these determine individual health behaviours [36]. Yet, wider macro social and structural factors such as economics, law enforcement, culture, religion, and politics also affect peoples’ health behaviours [29], including drug use [37].

The effects of macro-structural factors on health behaviours are particularly prominent in regard to HIV [35, 38]. In this regard, Baral et al. [35] argue that understanding and addressing the wider scope and multiplicity of determinants of HIV risks is essential to the control of the HIV epidemic. Indeed, existing studies exploring root causes of HIV risk suggest that common underlying determinants of HIV risks among injecting drug users and other key populations include individual biomedical factors such as circumcision, behavioural factors such as health seeking, and structural factors such as policing and health systems’ capacity [15, 35, 39]. For example, aggressive or discriminatory policing of marginalised or criminalised populations such as injecting drug users can cause them to share used needles, as they avoid to get arrested while obtaining clean needles [40, 41].

Thus, elaboration of HIV behaviour among women should capture broader ecological forces that influence their behaviours, taking into account potential resources, risks, and protective factors located within their communities and wider macro-environments. Besides identifying influences of health behaviour, the social ecology theory is also useful in the identification of potential solutions to ill health [32, 34], a function that is particularly suitable for the present study and its purpose of discussing potential implications on services, policy, and research to mitigate HIV risks among women who inject drugs. The use of social ecology theory is also relevant in Kenya where harm reduction services are currently in development [24], and where—as we argue in this paper—the current harm reduction policy has predominantly focused on clinical interventions, with relatively limited focus on structural interventions. In this context, there is an opportunity for developing additional interventions for women who inject drugs, based on women’s social structural ecologies and drivers of risky behaviours therein.

### Ethical approval

This secondary analysis was approved by the ethics committee of the Faculty of Health and Medicine at the University of Lancaster (ref: FHMREC16082). The ethics committee of the Kenya National Commission for Science, Technology and Innovation approved the primary study protocol (ref: P/15/8861/4510).

## Results

### Drug-use and sexual profile of participants

As shown in the following Table 1, 96% of the participants had ever been tested for HIV. Over a fifth (22%) of the participants were HIV positive. Condom use among the sample was generally low: a third (31%) of participants were consistent condom users, a third (29%) were inconsistent condom users, and another third (31%) had never used condoms. Seven of the ten participants living with HIV were inconsistent condom users, while the others used condoms always (*n* = 1), was not sexually active (*n* = 1), or did not provide that information (*n* = 1). Furthermore, 29% of the sample had drug-using partners. Of these, 11% reported that their main sexual partners were drug injectors, while 18% had partners who smoked or snorted drugs. Notably, 20% (*n* = 9) reported that their main sexual partner did not use any drugs. Furthermore, 29% reported a history of being sexually assaulted, and 48% had been exposed to physical and other forms of violence (Table 1).

**Table 1:** HIV testing and sexual characteristics of the study sample

Characteristic	IDI	FGDs	Total	Percent
HIV testing				
Ever tested				
Yes	23	20	43	96%
No	0	1	1	2%
Unknown	1	0	1	2%
Last tested				
In last month	2	5	7	16%
1–3 months	13	9	22	49%
3–6 months	3	2	5	11%
Over 6 months	0	0	0	0%
Over 1 year	5	4	9	20%
Unknown	1	0	1	2%
N/A	0	1	1	2%
Where tested				
Outreach	4	9	13	29%
Hospital/clinic	12	6	18	40%
Drop-in centre	5	4	9	20%
Prison	2	0	2	4%
Unknown	1	1	2	4%
N/A	0	1	1	2%
Collected results				
Yes	23	19	42	93%
No	0	1	1	2%
Unknown	1	0	1	2%
N/A	0	1	1	2%
HIV status				
Positive	7	3	10	22%
Negative	13	15	28	62%
Unknown	1	2	3	7%
Not willing to disclose	3	1	4	9%
Main sexual partners drug use				
Injecting	4	1	5	11%
Smoking	4	4	8	18%
None	6	3	9	20%
N/A	9	13	22	49%
Unknown	1	0	1	2%
Last time had sex				
Last 7 days	10	3	13	29%
1–4 weeks	3	4	7	16%
1–3 months	3	4	7	16%
> 3 months	5	10	15	33%
Not disclosed	2	0	2	4%
Unknown	1	0	1	2%
Condom use				
Always	9	5	14	31%
Sometimes	4	9	13	29%
Never	10	4	14	31%
Not disclosed	0	3	3	7%
Unknown	1	0	1	2%
Sexually harassed				
Yes	6	7	13	29%
No	16	14	30	67%
Not disclosed	1	0	1	2%
Unknown	1	0	1	2%
Sexually assaulted				
Yes	2	11	13	29%
No	17	10	27	60%
Not disclosed	1	0	1	2%
Unknown	1	0	1	2%
Other violence				
Yes	11	11	22	49%
No	11	10	21	47%
Not disclosed	1	0	1	2%
Unknown	1	0	1	2%

*Abbreviations*: *IDI* in-depth interview, *FGD* focus group discussion, *SD* standard deviation

Results showed that HIV risks among the sample fell into two broad categories: sexual and injecting-related. In keeping with the socio-ecological framework, identified influences of HIV risks were mapped onto individual, interpersonal, and societal-structural domains.

### Individual-level influences of risky behaviours

#### Low-risk perception

Throughout IDIs and FGDs, women reported that they received health education related to HIV and harms of drug use from outreach workers. Participants stated that outreach workers ‘*educate us*’ regarding ‘*infections*’ (IDI participant, aged 24, Mombasa) and ‘*how to stop drug addiction*’ (IDI participant, aged 26, Kilifi). Indeed, nearly all of the drug users had been contacted by outreach workers prior to the study and seemed to be generally aware that sharing of needles increased the risk of acquiring HIV infection. Being cognizant of that sharing of needles was to be avoided, some IDI participants seemed defensive when asked whether they shared needles, with one claiming that ‘*everybody uses their own needle*’ (IDI participant, aged 32 years, Kilifi). Despite this assertion, however, data from FGDs revealed that sharing of syringes and needles was common. While IDI participants denied sharing injecting equipment with others, which could be due to social desirability bias, participants in focus groups openly described how needles were ‘*going around*’ or ‘*travelling*’ from person to person (FGD participant, Mombasa).

Although women were more emboldened to acknowledge the presence of needle-sharing in a group context, some sought to distance themselves from this practice, suggesting that it was others that practised it, and not themselves. As an illustrative example, one participant claimed that ‘*there are others who, if it is night and she gets a used needle on the road, and she is injecting, she would re-use it*’ **(**FGD participant, Mombasa). Despite this kind of self-distancing and ‘othering’, focus group participants agreed that sharing of injecting equipment was taking place ‘*currently*’ (FGD participant, Mombasa). They stated that this practice was the reason why HIV was common among themselves:

You find that one needle travels among approximately seven people. This issue is contributing a lot to HIV (FGD participant, Mombasa).

Regardless of whether they attempted to conceal it or not, participants’ narratives indicated the presence of a disconnect between their attitude, knowledge, and actual practice related to sharing needles. While they were clearly aware of the dangers involved, they continued to share needles on an ongoing basis. Exploration of the contradiction between knowledge and practice suggested that participants’ sharing of needles was due to a somewhat situational low perception of risk. For instance, one participant stated that her peers ‘*think that the other person does not have the [HIV] virus, you say the needle is from your fellow, and that is why you see these issues of many infections come in*’ (FGD participant, Mombasa).

#### Intersection of trust and gender norms within intimate partnerships

Apart from being the underlying cause for needle-sharing, a low perception of risk also occurred within intimate partnerships. Due to this situational low-risk perception, six women had regularly participated in sexual activities that increased their risk of acquiring infections from their husbands and regular partners. As shown in Table 1, several women had stable partners who were also injecting drugs, mostly heroin. However, despite their intimate partners being at high risk of HIV on account of injecting drug use, women were not deterred from having unprotected sex with them, as might be noted in the following excerpt:

Q: When was the last time you had intercourse when high on drugs?

R: Last week.

Q: Explain to me how it was.

R: I was with my boyfriend, we just had it as usual, and we get intimate without a condom as I have no worries at all.

Q: Is your boyfriend also a drug addict?

R: Yes.

Q: How does he use drugs?

R: He injects himself (IDI participant, aged 30, Kilifi).

As may be deduced from above exchange, women routinely participated in unprotected sex and drug use during sex with their partners, due to a perception that their partners were uninfected. As such, the above women perceived no reason to worry. Other participant’s narratives indicated that this low-risk perception was deeply intertwined with women’s trust of their sexual partners. For instance, in response to a question regarding when she had last injected heroin during sex, a woman who had reported that she never used condoms stated as follows: ‘*yesterday but one, when I was with my husband, the man whom I can trust*’ (IDI participant, aged 36 years, Kilifi).

Despite trust of sex partners playing a prominent role, the extent to which a couple’s sexual behaviours were under the full control of the women themselves was not always clear. Nevertheless, our findings showed that some participants were inadvertently getting exposed to HIV while in the pursuit of fulfiling perceived ideals of a good sexual relationship with their men, whom they were expected to trust. A number of participants reported feeling compelled to take drugs in order to facilitate good sex with intimate partners, especially when they were not in the mood for it, or after they had argued:

Maybe you have differed, and for a long time you are not in the mood. You do not have the feelings. So you see, your partner forces you to use. He tells me “hold these two, one for you to inject and the other I will inject, then we will [have sex]” (IDI participant, aged 21 years, Mombasa).

As evident in the above excerpt, there were implicit and opposing interplays between women’s cognition and their behaviours with their sex partners. For instance, the above excerpt implied this participant would not normally take drugs during sex, except to fulfil an expected sexual role. As was the case among most married or cohabiting women, the pursuit of expected sex with her partner also meant that this participant would not use a condom with him. Referring to her husband, another woman mentioned that ‘*whenever he comes home he brings some [drugs] along for me. I have to take drugs for me to feel pleasure. Without drugs, it is like you are forcing me*’ (IDI participant, aged 36 years, Kilifi). As might be noted here, an inequitable cathexis and gender structure dictated that women were obligated to have sex with their men and often to inject prior to sex as sex would otherwise be undesirable. Suggesting that women were not adequately empowered to control their own exposure to risks, stakeholders gave examples of how intimate partners wielded powerful external influence over women. In this context, ‘*bonding with spouses*’ was blamed for making women ‘*go back to risky behaviours*’ (CBO Program Manager, Kilifi). In sum, risk perception, inequitable gender norms, and trust intersected to potentially expose these women to HIV.

### Interpersonal influences of risky behaviours

#### Perils of transactional relationships and sex work

In contrast to the low perception of risk of acquiring HIV infection from their husbands and regular partners, women who engaged in transactional relationships, whereby sex was exchanged for a variety of resources, recognised that they were at high risk of HIV. Overall, a smaller proportion of 18% of the participants were married, while 53% were single and 27% were cohabiting. Because single or cohabiting women tended to be in transactional sexual relationships, transactional sex was more prevalent among the sample as a whole, despite it being perceived as risky. Indeed, most single women regularly exchanged sex for drugs, protection from the police, and accommodation. In an illustration of how this typically occurred, a participant explained that ‘*there are young men who usually sell. Sometimes you have lacked [drugs], and he sees you. He will tell you ‘have sex with me then I will give you a sachet. You can have 1 or 2 sachets, as long as you make me happy’, so you have to accept*’ (IDI participant, aged 26 years, Mombasa). In addition to a number of other similar accounts from six other women, several stakeholders also claimed that a large number of women were involved in transactional sexual relationships with peddlers:

To sustain the addiction behaviour, they have to make friendly relationships with peddlers if they are going to get free drugs. Free in quotes. They have to give their bodies to the peddlers for them to get drugs (CBO Program Manager, Kilifi).

In this context, it was usual that ‘*when you have a relationship with someone like that, and he sells, he provides you with drugs so that you can make him happy [sexually]*’ (IDI participant, aged 26 years, Mombasa). Besides getting heroin from her peddler boyfriend, this participant also explained that she also gained a significant amount of social capital from her boyfriend in exchange for sex. This social capital was created by the extensive social networks that most peddlers had, sometimes with the police. Explaining that peddlers were well connected socially, she explained that ‘*in other ways, he protects me from the bad things that happen at the drug dens. He will be the first person to be informed if the police are arresting people. He tells me to leave, or we leave together. He cannot leave me to be arrested, you see!*’ (IDI participant, aged 26 years, Mombasa). Despite these kinds of benefits, women who engaged in transactional sex were highly vulnerable to abuse by their sexual partners. Women’ accounts suggested that those who had transactional sexual partners were regularly raped by them, as might be deduced from the following quote:

There is a guy who used to accommodate me because I could not afford to pay for a house. I used to see as if he used to force me to have sex with him, it’s like he used to rape me…as in, I did not like it. However, because it was rent, it was like a type of rent, it was compulsory for me to give it by way of sex. So I had to accept (IDI participant, aged 33 years, Kilifi).

Our data suggested that women who were engaged in transactional sexual relationships primarily to acquire for drugs or accommodation tended to maintain the same partner for some length of time. However, the most common form of transactional sexual relationships took the form of sex work, where sex with multiple casual partners was exchanged for money. Sex work was the main occupation among 13 women, comprising 29% of the sample. Besides it being highly prevalent, some women reported having sex with more than ten men daily. Discussing the high turnover of their clients, one sex worker stated that ‘*regarding men, we exchange with them like clothes*’ **(**FGD participant, Mombasa). Other participants illustrated the multiplicity of their sex partners by describing their sex life using phrases such as ‘*I roam about*’ (IDI participant, aged 21 years, Mombasa), ‘*I move here and ther*e’ or ‘*I move like a bird*’ (IDI participant, aged 26 years, Mombasa). Besides multiplicity of sexual partners, specific situations in which women were potentially exposed to infections were described:

To tell the truth, whenever I have no money to buy drugs, I go and sell my body. At times when I go for prostitution, accidents happen, like a time when the condom burst. In another incident, I had a client, but I realised that I did not have a condom, but because I needed the money, I performed a blowjob without a condom (IDI participant, aged 30 years, Kilifi).

#### Drug use during sex work

The above sexual risks were aggravated by injecting of heroin during sex work. Engaging in sex while high on drugs, colloquially referred to as ‘being steam’, was seen as essential by almost all the 13 women involved in sex work in our sample. They claimed that injecting drugs during sex work enabled them to bear the shame of it:

I usually have sex when I am steam. It is very hard for me to have sex when I am sober because when I do sex work, I am in business, it is not something that I wish to do. So I have to use drugs and be steam so that I am not shy when I do sex work (IDI participant, aged 26 years, Mombasa).

However, because most participants regularly lacked money to purchase drugs, they frequently asked their prospective clients to buy drugs for them prior to sex, which further exacerbated the risks associated with drug injecting and sex work by weakening their ability to bargain for safer sex:

If a man wants to have sex with me and at that time I have not injected, then I tell him “I cannot go with you because I have not injected yet”. He then he takes me to get drugs, but I am obliged to have sex with him. We use the drugs together, then he uses me later (IDI participant, aged 30 years, Kilifi).

#### Intersections of sex work, gender violence, and power

As might be noted above, participants felt disempowered and commoditised, particularly because they were unable to negotiate for condom use when intoxicated. Furthermore, our data suggested that once clients bought drugs for them, women were left even more vulnerable to sexual violence at the hands of their clients. Indeed, a third of the women, most of who were involved in sex work, had experienced sexual harassment or assault (Table 1**)**. Accounts from six of these participants showed that being cajoled to have condomless sex was the norm, and this often morphed into rape or physical violence:

The work we do… we are at high risk. Sometimes you go with someone, and he is rough, or wants to use you and does not want to pay you. If you resist, you are beaten. He tells you "I want to have sex without a condom." If you refuse, he will bring chaos (IDI participant, aged 24 years, Mombasa).

Nearly half of the women reported having experienced some form of violence, usually after they did not yield to demands for condomless sex from clients. Commenting on the regularity of rape, one sex worker who often had sex while high on heroin described a recent incident of her own:

I had injected myself such that I did not know myself. The following morning is when I found out that I had been raped. Do you understand? I realised that I did not have clothes, I could not do anything, I could not even walk, I was bleeding. Afterwards, outreach workers from REACHOUT came to pick me and took me to the hospital. By good luck, I was tested for HIV and found that I did not have it (IDI participant, aged 21 years, Mombasa).

As illustrated in the preceding excerpts, participants who engaged in sex work were aware of the risks associated with it, such as HIV. Clearly, most of them preferred to use condoms with their sex work clients, with one stating that she would insist that ‘*they pay me for sex but using a condom*’ (IDI participant, aged 26 years, Kilifi). Another stated:

Yes, sex work is my job, but even if it is my job, I always protect myself so much. I usually have sex with someone using a condom; if there is no condom, I do not have sex (IDI participant, aged 21 years, Mombasa).

Despite being cognizant of the risks, data suggested that these women were frequently precluded from practising safer sex. One sex worker narrated how she used to insert male condoms into her vagina in an attempt to minimise potential risks, having little power to ensure that her clients used condoms:

They insist that they do not want to use condoms, so at times I agree because of arosto [withdrawal], but I take the male condom, tear it and insert it into my vaginal tract, so that when he ejaculates inside, the sperms do not penetrate through and bring infections. I do not know if this helps or not? (IDI participant, aged 30, Kilifi).

### Societal-structural influences of risky behaviours

#### Economic influences of risky behaviours

In spite of sex workers’ determination to protect themselves from sexually transmitted infections, economic considerations compelled them to abandon their condom use. Describing a typical scenario where this occurred, an FGD participant stated that:

Another one tells you, "I have one thousand shillings, but I don’t want to use a condom, but if you want sex with a condom, I will give you two hundred shillings." Now you are compelled; seeing that the one thousand is a lot, you are forced to do it without a condom (FGD participant, Mombasa).

Furthermore, data suggested that most participants entered into sex work due to joblessness and poverty. Five women of varying ages blamed lack of jobs for their entry into sex work, with one stating that ‘*sex work has been my main means of getting money to use*’ (IDI participant, aged 26 years, Mombasa). Another lamented that ‘*[Because] I don’t have a job, I sleep with any man as long as he has money*’ (IDI participant, aged 33 years, Kilifi). Given the fact that most of her peers were jobless, another participant suggested that sex work was inescapable, claiming that ‘*It behoves to engage in sex work*’ (IDI participant, aged 30 years, Mombasa).

Despite the economic benefits, women rued the fact that sex work exposed them to HIV, yet it was nearly inescapable as they needed income. To illustrate this, one participant described ‘*sucking in blowjob*’, ‘*condoms bursting*’, ‘*having sperms in my mouth*’, and other forms of accidents, but she quickly added that ‘*I didn’t mind if there are any risks because I was in need of the money for buying drugs*’ (IDI participant, aged 30 years, Kilifi). Others in FGDs rued participating in unwanted sexual practices, yet they felt forced by economic circumstances to engage in it. Supporting comments from other FGD participants, one sex worker narrated how her clients would often demand unprotected anal sex, stating '*they insist*: "*I don’t want in front, I want behind*"*. In other words, he forces you, but you want that money, so you are forced to close your eyes*' (FGD participant, Mombasa). Even when women had the opportunity to use condoms with clients, they stated how economics caused them not to use them. In theory, outreach workers were supposed to distribute free condoms, but there were times when women had run out of them and needed to purchase them. Here, economics barred these women from practicing safe sex, as explained by the following participant, who was also HIV positive and was keen to use condoms:

There are times we lack free condoms, and you are forced to buy, but at times it is hard: you have come onto the street, and you do not even have 10 shillings to buy one. That is where the problem comes in (IDI participant, aged 26 years, Mombasa).

#### Inequitable gender expectations and vulnerabilities

Indeed, and perhaps because they were aware of the risks involved, women often took upon themselves the responsibility of ensuring that a condom was used during sex work. Given their financial impoverishment, however, they were generally unable to avail condoms. Despite being able to afford condoms, their male clients normally preferred condomless sex, however, and regularly took advantage of women’s lack, as explained by the above sex worker who was living with HIV:

He will take that opportunity and say “it is not my fault that you don’t have it, so let’s proceed” (IDI participant, aged 26 years, Kilifi).

This kind of situation may have created conditions for clients to acquire HIV from infected sex workers. These finding suggested that the presence of differential gender responsibilities and expectations related to the provision of condoms created opportunities for HIV transmission, fueled by economic inequalities. In effect, these gendered economic inequalities made women more vulnerable, as they were often unable to provide condoms, and control their risks.

Underscoring the issue of gender vulnerability was the observation that most of the peddlers at the study towns were male: only three of the 45 women in our sample were peddlers. Women were regularly exploited by male peddlers who were known to have multiple transactional sex partners and to use drugs too. Thus, the predominance of men in peddling added a further vulnerability to the gendered risks that confronted women, since peddlers had an upper hand:

You can go to borrow drugs on credit, and they tell you "what use is it to borrow? If you can sleep with me, I will give you even more." But because you have arosto [withdrawal] you are forced to do as he wishes (IDI participant, aged 30 years, Kilifi).

Just like other male clients of sex workers, male peddlers frequently demanded that transactional sex was condomless. One participant who was HIV positive (mentioned previously) explained that ‘*normally at the drug injecting dens, where someone gives you drugs in exchange of sex, mostly we don’t use condoms*’ (IDI participant, aged 26 years, Mombasa).

Indeed, a program manager at a community-based organisation noted how the convergence of addiction and the possibility that women could always engage in sex work increased their exposure to infections, asserting that, ‘*women are vulnerable because of sex work and their need to use drugs*’ (CBO Program Manager, Kilifi). Thus, although women seemed to benefit economically from their transactional relationships with peddlers and other partners, this benefit was paradoxical in that it harmed them in other ways. In the words of this stakeholder, ‘*women see the peddlers and the spouses as their main backbone of support although in the real sense they are not*’ (CBO Program Manager, Kilifi).

#### Intersecting gender, economic, and health system influences

While the emphasis here was on the women’s economic needs, these influences occurred alongside others, including interpersonal influences. For example, women reported that they were compelled to engage in sex work to support their own and their partners’ drug use. In situations when men lacked drugs, they regularly required their women to ‘*go and hustle*’ (IDI participant, aged 30 years, Mombasa). Similar to three others, this participant was cognisant of her partner’s economic dependency on her, brought about her gender-related ability to engage in sex work, asserting that ‘*I am the one he is using as a means of survival*’ (IDI participant, aged 30 years, Mombasa). Another participant who had been involved in transactional sex for accommodation (mentioned previously) narrated how her partner (who was a cocaine user) also required her to get drugs for him, in addition to sex in exchange for accommodation. Noting the paradoxical shift, she narrated stressed that:

So it became I who was hustling. I could go to him for accommodation, but he did not have any means of getting drugs. He too stays and waits for me, it as if he were my child. So it’s as if I am paying him through sex and by looking for what he will use for buying his cocaine. And it’s been a daily routine. I feel very annoyed, but I have no other choice (IDI participant, aged 33 years, Kilifi).

Thus, economic pressure, and a reluctance of male partners to cater for themselves and their spouses, intersected with gender vulnerability to enhance risks that women were exposed to*.* Indeed, an outreach worker highlighted the confluence of these influences:

If a woman is into drugs, the risk is high, because they have double or multiple issues. They can do drugs, they can have a sexual partner who is a drug user, and at the same time, they might be involved in sex work with multiple other sex clients as well. So the risk is very high (CBO Outreach Worker, Mombasa).

**Fig. 1:**
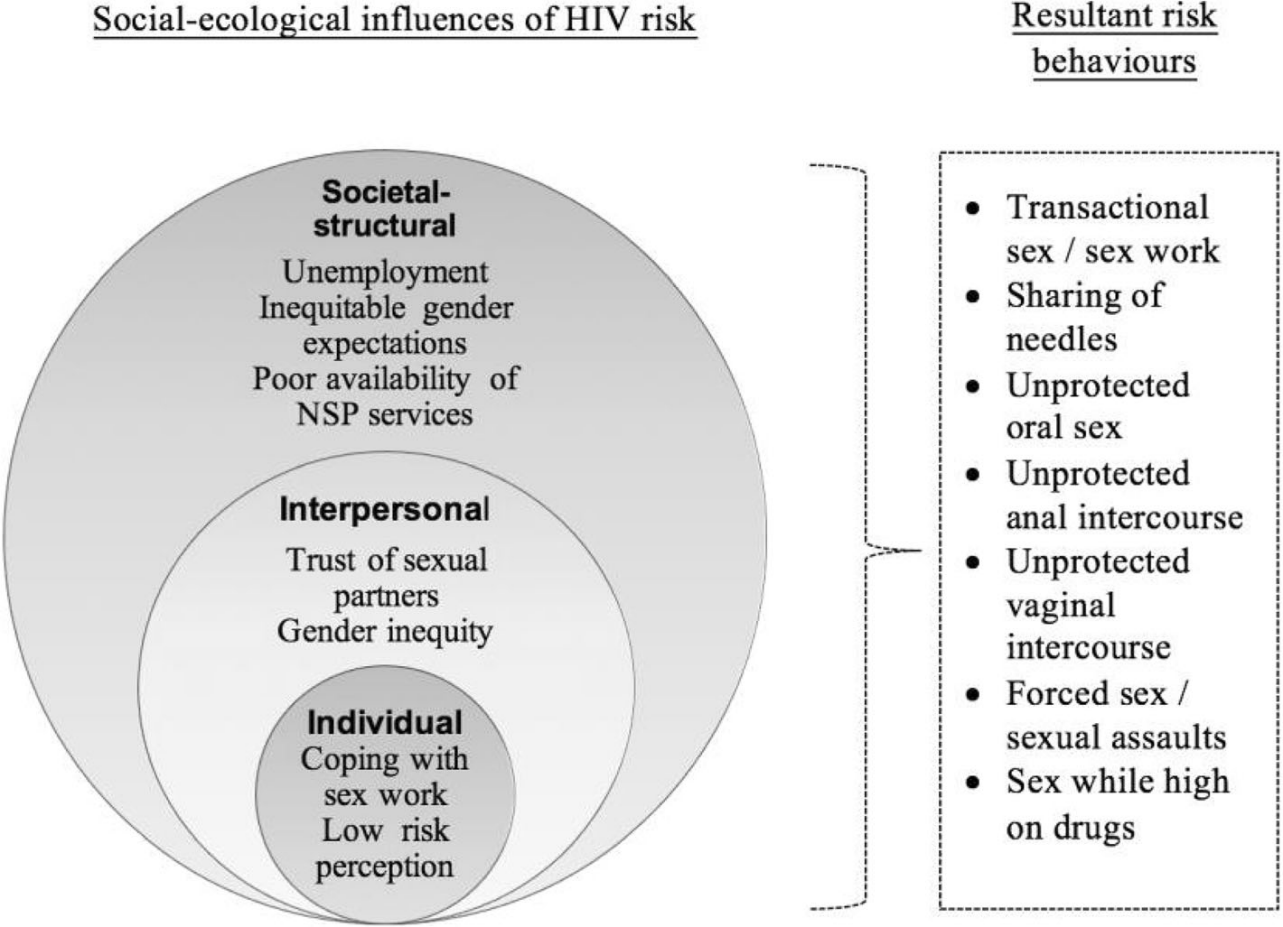
Determinants of risky injecting and sexual behaviours among women who inject drugs. HIV risks were produced by intersecting influences in women’s social ecologies

Underscoring the issue of intersecting influences was the observation that apart from propelling sex work, economic factors intersected with health system factors to increase unsafe injecting practices. Asked to provide specific situations in which sharing of needles took place, a focus group participant explained that needle sharing frequently occurred ‘*at night*’ when PWID commonly run out of clean needles (FGD participant, Mombasa). Another FGD participant elaborated that in these situations, women’s decisions regarding sharing needles depended on ‘*whether the chemist is still open*’ and whether they had ‘*money to buy [syringes]*’ (FGD participant, Mombasa). Due to their economic impoverishment, however, few could afford to purchase injecting equipment. Hence, although outreach workers did provide these commodities freely, they were inadequate to meet women’s needs throughout the day and night. This poor health services organisation intersected with economic pressures to produce unsafe injecting practices. This observation of intersection was consistent with patterns observed in regard to other drivers of risky sexual behaviours. In sum, women encountered diverse HIV risks emanating from their sexual and injecting behaviours, which were produced by intersecting influences in their social ecologies as shown in Fig. 1**.**

## Discussion

This paper reports HIV risks encountered by female injecting drug users in the Kenyan coastal towns of Mombasa and Kilifi. An important contribution of our study is in elaborating the social contexts of injecting drug use and attendant HIV risks among a relatively understudied population of female injecting drug users in Kenya. In our study, HIV risks among women who inject drugs fell into two broad categories: sexual and injecting-related. Our sample of women was regularly exposed to potential HIV infection via unsafe sexual and injecting practices. Not surprisingly, a fifth of them were already infected with HIV at the time of the study. At the most primary level, our study demonstrates the range of behaviours that expose women to HIV, including unsafe injecting practices, unprotected oral, vaginal and anal sex, sex while high on drugs, forced sex/rape, sex work, and other forms of transactional sex.

With the possible exception of having sex while high on injected drugs, the rest of these risky injecting and sexual practices have been widely documented in other studies. For instance, in Vietnam, Malaysia, and Tanzania, several studies have reported unprotected vaginal, oral, and anal sex as well as unsafe injecting among female injectors, in the context of intimate sexual relationships and sex work [42–44]. Sex work is particularly determinant of HIV acquisition among female injectors [45]. Despite the consistency of our findings with the above studies, our study makes a novel contribution to literature by documenting the practice and gendered motivation of having sex while high on drugs among local women who use drugs. While accounts of people engaging in sex while intoxicated with drugs exist in the literature, these typically feature men who have sex with other men [46, 47] and these predominantly use oral party drugs such as mephedrone, crystal meth (ecstasy), or gammahydroxybutrate (GHB), rather than opioids [48–51].

Here, our study distinguishes the motivation for having sex while high on drugs among our female sample to that documented among gay men. While the primary motivation for this practice among gay (and potentially other) men is to enhance sexual performance and pleasure [52], it was primarily used by the women in our study to cope with the shame and rigours of sex work. A few participants in our study also injected drugs to enable them fulfill their sexual obligations to their intimate partners. As such, our study extends existing literature by distinguishing the gendered purpose of drug use during sex by showing how motivation for this practice differs by gender. This divergence notwithstanding, our study’s findings are similar to those conducted among gay men, in showing that drug use during sex was linked with a limited use of condoms. Given the prevalence of sex work and its association with poor condom use among women who inject drugs in our study, the use of drugs during sex should be considered an important HIV risk behaviour locally. This is particularly relevant given that injecting drug use is becoming more prevalent in Kenya [9], and interventions for PWID are still nascent [24].

A critical finding from our study was the convergence and intersection of underlying social-ecological determinants of HIV risks. Unsafe sexual and injecting were produced at the intersection of low-risk perception, inequitable gender norms and power, economic pressures, and poor availability of condoms and needles. Risky sexual behaviours were natural exigencies of injecting drug use in the context of scarce economic resources, supporting claims that epidemics of substance abuse and HIV occur as an intertwined and synergistic phenomenon, often mediated by sexual violence [53]. For example, both scarce economic resources and inequitable gender power determined ways in which sex work increased HIV vulnerability, for example by making it condomless or laden with sexual violence. At the same time, these risks were aggravated by an inadequate supply of condoms due to poor health service organisation.

In sum, individual, interpersonal, and societal-structural determinants intersected to fashion HIV risks that women were exposed to in the course of their injecting drug use. This intersection of multiple drivers of risk created an environment laden with opportunities for women to acquire or transmit HIV and is consistent with the presence of an ‘HIV risk environment’ within the study context. Not surprisingly a fifth of the women were already infected with HIV. This convergence of determinants of HIV risk is consistent with Rhodes’s [54] assertion that ‘harm from drug use is contingent upon social context, comprising interactions between individuals and environments’.

### Implications for practice, policy, and research

Given the findings of multiple intersecting determinants of HIV risks, a combination of individual, interpersonal, and structural interventions would be needed to mitigate them. From a theoretical perspective, the assumption is that ‘multilevel interventions should be most effective in changing behaviour’ [29]. As such, regardless of their specific nature, interventions to mitigate HIV risks should as much as possible be combined, bearing in mind that just as determinants of risky behaviour intersect with each other, interventions are also likely to reinforce each other. Table 2 summarises some of the interventions that we suggest could be implemented in a combined fashion to mitigate the various risks identified in our study.

**Table 2:** Determinants and potential mitigations of HIV risks

Social-ecological domain	Determinants of HIV risk	Potential mitigation
Individual	• Coping with sex work	• Self-efficacy training
	• Low-risk perception	• Risk assessment skills
Interpersonal	• Inequitable gender power	• Couple counselling
	• Trust of sexual partners	• Gender transformation
Societal structural	• Poor availability of NSP services	• Expansion of NSP services
	• Unemployment/poverty	• Livelihood and employability interventions

#### Individual-level interventions

At the individual level, focusing on self-efficacy, rather than just on education regarding risks as is the case currently, is required. Our findings show that despite being aware of various HIV risks, participants’ behaviours were incongruent to their knowledge. Besides the notable diversity and co-occurrence of HIV risks, an important feature was the involuntary way in which they were experienced by women, in a context of inequitable gender power and norms. Thus, focusing on individual and collective self-efficacy could impart actual skills and competencies to enable women change their injecting behaviour or avert potential sexual violence. These suggestions are supported by research showing that improved self-efficacy reduces adolescent substance abuse [55]. Strengthening women’s self-efficacy is particularly relevant given that they were often involuntarily exposed to HIV due to inequitable gender power. For the most part, married and cohabiting women placed significant emotional importance on their relationships and attendant gender expectations, while those in transactional sex were insufficiently empowered to control their exposure to sexual risks imposed on them by men. Even when sexual and injecting risks were understood by the women, both economic and emotional considerations within intimate relationships and sex work tended to override concerns regarding potential infection. These dynamics clearly affected both women’s HIV risk-taking and risk management and supports our suggestion that strengthening women’s efficacy to behave based on their assessment of risk is essential.

#### Interpersonal interventions

At the same time, it is nearly impossible to adequately empower these women to adopt safer sex and injecting practices without addressing both the gender and economic drivers of their risky behaviours. As shown in this and other manuscripts from our sample [56], intimate sex partners wielded significant influence on women’s sexual and injecting behaviours, which need to be addressed. To start with, adopting a couple-based approach to reaching injecting drug users would be useful in mitigating inequitable gender power, by creating opportunities for couple counselling regarding dangers of drug in the context of condomless sex. Existing research shows that couple-based approaches are effective in preventing HIV because of their ability to transform micro-social contexts of HIV risks among PWID in intimate relationships [57, 58]. For those who have a low-risk perception, interventions to faciliate objective risk identification would be useful, as also proposed in India [59]. Because gender norms that encourage men to make decisions for their families are prevalent in the study context [56], additional interventions to transform inequitable gender norms should complement couple-based approaches. For example, strategies such as community conversations that focus on educating wider communities can provide a good ground for dismantling harmful gender norms which seemed to create expectations that only women are responsible for safer sex. Community conversations have been applied to transform reproductive health behaviours in the study context [60].

#### Societal-structural interventions

Given the consistency of our findings with Rhodes’ [54] concept of ‘risk environments’, an important recommendation from our study is that upstream structural interventions for female injectors should be incorporated into harm reduction services. Currently, and similar to the situation in other countries [35], harm reduction services in Kenya tend to over-rely on individual and interpersonal interventions while ignoring structural interventions. The National Guidelines for the Comprehensive Management of the Health Risks and Consequences of Drug Use prescribe the harm reduction package recommended by the World Health Organization [14], with practical emphasis on the provision of clean needles, education, and methadone [13]. The National Protocol for Treatment of Substance Use Disorders also emphasises downstream services such as clinical treatment and outreach [61]. These recommendations are of course relevant given our findings showing that a need for clean needles and condoms exists. However, less thought of influences such as joblessness, gender inequalities in economic power, and a lack of accommodation, were also influential.

Therefore, to adequately mitigate injecting and sexual HIV risks among women, it is essential to address the underlying gender and economic determinants. For example, promoting women’s financial independence should be an essential part of HIV prevention. Incorporating livelihood, employability, microfinance, and low threshold housing into harm reduction could cushion women from the negative influences of their intimate partners [56]. Existing evidence shows that women injectors who are better off financially through livelihood and microfinance interventions are more able to better control their condom use, needle sharing, and numbers of sex work clients [58, 62].

At the same time, strengthening health systems is needed to ensure universal accessibility of clean needles, including at night when sharing of used needles was reported to be prevalent. Instead of PWID having to buy syringes from pharmacies, allowing free distribution of syringes at pharmacies could be explored as an alternative service and policy option. Free distribution of syringes via pharmacies, together with the provision of methadone, residential drug treatment, and designated safe-injecting spaces (shooting galleries) feature in countries with advanced harm reduction services [63]. As such, policy shifts will be required in Kenya to integrate macro-structural interventions while ensuring universal coverage of downstream harm reduction services. Both micro- and macro-interventions should be provided in an integrated fashion, incorporating the recommended package of harm reduction and HIV services as emphasised by others [64, 65]. Indeed, the social-ecological approach utilised in this study challenges the idea that determinants of drug use operate in isolation and therefore asserts that interventions should be interconnected or linked [33].

### Limitations

Participants in our study were recruited via two community-based organisations. As such, our findings may not reflect the experiences and perspectives of women who are not in contact with such community-based harm reduction services. In addition, similar to other studies of drug use [66], social desirability bias may have affected our findings, given the sensitive and personal nature of injecting and sexual risks that the IDIs and FGDs involved. Furthermore, this study may still be affected by interpretation bias stemming from the background and experiences of the coder (GM). Because this secondary analysis was based on PhD studies, it was not possible for more than one researcher to code the transcripts. However, the use of an established social-ecological theoretical framework to guide the interpretation, combined with rigorous and transparent coding methodology, mitigated potential bias. Despite residual limitations that may exist, this study contributes to a better understanding of women’s injecting contexts, which can inform both gender-sensitive interventions and future studies.

## Conclusion

Although women constitute a third of all drug users globally [67], they are disproportionately underrepresented in studies of drug use [23], including in Kenya, where previous study samples have been predominated by men. Our study successfully advances the application of secondary analysis in qualitative research to elaborate determinants of HIV risks among women who inject drugs, using the social ecology theory. Participants were highly vulnerable to acquiring HIV infection via sexual and unsafe injecting practices, and a fifth of them were already infected with HIV. Findings show that women’s risks to HIV should be viewed broadly as a product of their social ecologies, extending from their personal circumstances, relationships, and social-structural contexts. These findings support assertions that HIV and other harms of injecting drug use are deeply embedded in the injectors’ environment. This environment created opportunities for women to acquire or transmit HIV and is consistent with the presence of an ‘HIV risk environment’ within the study context. To address the convergence of determinants of HIV risks, a combination of interventions spanning women’s ecology should be central to harm reduction and HIV programs, with emphasis on upstream interventions that have hitherto received limited attention.
